# Divergent airway microbiomes in lung transplant recipients with or without pulmonary infection

**DOI:** 10.1186/s12931-021-01724-w

**Published:** 2021-04-23

**Authors:** Lisa I. Påhlman, Lokeshwaran Manoharan, Anna Stjärne Aspelund

**Affiliations:** 1 Department of Clinical Sciences Lund, Division of Infection Medicine, Lund University, BMC B14, 221 84 Lund, Sweden; 2 Division of Infectious Diseases, Skåne University Hospital Lund, Lund, Sweden; 3 Wallenberg Centre for Molecular Medicine, Lund University, Lund, Sweden; 4 Department of Laboratory Medicine, National Bioinformatics Infrastructure Sweden (NBIS), Lund University, Lund, Sweden; 5 Division of Infection Medicine, Hospital of Helsingborg, Helsingborg, Sweden

**Keywords:** Lung transplant recipients, Microbiome, Airway infection, Inflammation, Cytokines

## Abstract

**Background:**

Lung transplant (LTx) recipients are at increased risk for airway infections, but the cause of infection is often difficult to establish with traditional culture-based techniques. The objectives of the study was to compare the airway microbiome in LTx patients with and without ongoing airway infection and identify differences in their microbiome composition.

**Methods:**

LTx recipients were prospectively followed with bronchoalveolar lavage (BAL) during the first year after transplantation. The likelihood of airway infection at the time of sampling was graded based on clinical criteria and BAL cultures, and BAL fluid levels of the inflammatory markers heparin-binding protein (HBP), IL-1β and IL-8 were determined with ELISA. The bacterial microbiome of the samples were analysed with 16S rDNA sequencing and characterized based on richness and evenness. The distance in microbiome composition between samples were determined using Bray–Curtis and weighted and unweighted UniFrac.

**Results:**

A total of 46 samples from 22 patients were included in the study. Samples collected during infection and samples with high levels of inflammation were characterized by loss of bacterial diversity and a significantly different species composition. *Burkholderia*, *Corynebacterium* and *Staphylococcus* were enriched during infection and inflammation, whereas anaerobes and normal oropharyngeal flora were less abundant. The most common findings in BAL cultures, including *Pseudomonas aeruginosa*, were not enriched during infection.

**Conclusion:**

This study gives important insights into the dynamics of the airway microbiome of LTx recipients, and suggests that lung infections are associated with a disruption in the homeostasis of the microbiome.

**Supplementary Information:**

The online version contains supplementary material available at 10.1186/s12931-021-01724-w.

## Background

Lung transplantation (LTx) is an increasing treatment option for end-stage lung disease, but despite advances in modern medicine the mortality rates are still high with a median survival of 6.0 years [1]. LTx recipients are at high risk for airway infections due to heavy immunosuppressive therapy in combination with constant exposure to the external environment, micro-aspiration of the oropharyngeal microbiome, and defective mechanical defences due to denervation of the allograft, disrupted lymphatic drainage and impaired muco-ciliary clearance [2]. Previous studies on LTx patients have shown that airway infections are common, and that patients experiencing episodes of pneumonia have higher mortality rates [3]. It is therefore important to correctly diagnose and treat airway infections in this group of patients. Most previous studies on the aetiology of airway infections in LTx recipients have used results from culture-based techniques [3–5]. However, this approach has several drawbacks. For example, cultures are limited to known and culturable species, and they are unable to differentiate between microbes causing disease versus microbes colonizing asymptomatic carriers [6]. This was illustrated in an earlier study, where we demonstrated that bacterial findings in BALF cultures were similar in patients with and without clinical signs of infection at the time of sampling [7]. The difficulty in interpreting culture findings emphasizes the great need for new tools, including microbiome analyses, to identify and understand the aetiology of respiratory infections in these patients [6].

In this study, we analysed the microbiome composition of bronchoalveolar lavage fluid (BALF) samples collected from LTx patients during their first post-operative year. The findings were correlated with clinical signs of infection and inflammatory markers in BALF. The aim was to characterize and compare the airway microbiome composition during infection and non-infection.

## Methods

### Study setting and patient population

The samples included in this study are from a patient cohort described previously [8]. In short, adult patients accepted for LTx during the period October 2012 to December 2014 were eligible for inclusion. Patients under 18 years of age and patients with follow-up at other sites were excluded. Standard protocol for immune suppression included induction therapy with anti-thymocyte globulin followed by tacrolimus or cyclosporine, mycophenolate mofetil, and steroids. All study participants were followed for a maximum of 1 year after transplantation. BALF samples were collected at routine bronchoscopies at 3 and 6 months after LTx, and at diagnostic bronchoscopies in response to symptoms. BALF samples collected less than 7 days after the previous sampling were excluded from the study. All samples were analysed with routine bacterial cultures at the Department of Clinical Microbiology at Skåne University Hospital. The likelihood of pulmonary bacterial infection at the time of sampling was graded as no, possible, probable or definite infection based on (A) radiology, (B) macroscopic appearance at bronchoscopy and inflammatory cells in BALF, (C) clinical symptoms of airway infection, and (D) bacterial culture results [see Additional file 7: Table S1]. Samples classified as no infection had none of the infection criteria. Possible infection fulfilled one criterium. Probable infection had two or three criteria (A and/or B plus C and/or D). Definite infection fulfilled criteria A-D. The definition of infection was adapted from the ISHLT guidelines [9]. In this study, samples classified as probable and definite infection were considered having an infection, whereas no and possible infection were considered representing no infection. The inflammatory biomarkers Heparin binding protein (HBP), IL-1β and IL-8 were analysed in the BALF samples and cut-off values determined as previously described [8].

### Sample preparation and sequencing

BAL procedure followed a standardised protocol [8]. Study samples were obtained after instillation of 20 mL phosphate-buffered saline (PBS), where the initial 10 mL of recovered BALF were discarded after which a study sample of 10 mL was collected. BALF samples were centrifuged and total DNA was subsequently extracted from the cell pellet using QIAamp DNA Mini Kit (Qiagen, Hilden, Germany) according to the manufacturer’s protocol. Library preparation, fragment analyses and sequencing of the 16S amplicon (V3-V4) region were done by BaseClear B.V, Leiden, The Netherlands. In short, the 16S amplicon libraries were prepared from the DNA samples using 341F [CCTACGGGNGGCWGCAG] and 805R [GGACTACHVGGGTWTCTAAT] primer pair [10] with sample indexes. These amplicons were then analysed in FragmentAnalyser and the expected amplicon size (~ 434 bp) were selected and purified using BluePippin (2% gel) (Sage Science, MA USA) and AMPure beads. These purified samples were then pooled and sequenced with Illumina MiSeq paired-end (2 X 300 bp) sequencing.

### Bioinformatics and statistical analyses

The demultiplexed samples from MiSeq were checked for quality using FastQC [11] and processed with the QIIME2 (v.2018.11) [12]. The amplicon sequence variants (ASVs) for 16S were predicted using the DADA2 [13] pipeline within QIIME2. These 16S ASVs were then curated separately using LULU [14]. The taxonomy of curated ASVs were predicted using the tool VSEARCH [15] in combination with the SILVA (v. 132) database [16]. The resolution between the genera *Burkholderia*, *Caballeronia* and *Paraburkholderia* is not well defined in the SILVA-16S database [17]. This group of genera will further be referenced as “*Burkholderia*-group” in this manuscript. The alpha- and beta-diversity measures for these samples, including the phylogeny based measures (UniFrac [18]) were calculated in QIIME2. Further, all the statistical analyses on these samples were performed using the PHYLOSEQ [19] and ‘vegan’ [20] packages in R. For calculating the significant organisms that are abundant in different samples, DESeq2 [21] based differential abundance analysis was performed. Categorical data were compared using Fisher’s exact test.

## Results

### Patient cohort and samples

**Fig. 1:**
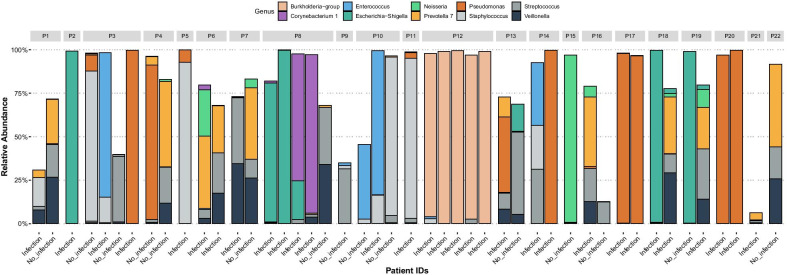
Microbiome composition of the study samples. The figure shows the microbiome of all included study samples from each patient. The patient number is indicated above the bars, and each bar shows the relative abundance of the 10 most common bacterial genera in this study. The clinical status of the patient at the time of sampling is indicated below each bar

In total, 46 BALF samples from 22 patients were included in the study, see Table 1 and [Additional file 8: Table S2] for patient characteristics. The study participants contributed a median of 2 samples per patient (range 1–4). Of these, 31 (67%) were collected during an airway infection episode, and 16 (35%) during ongoing antibiotic treatment (Table 2). The majority of samples collected during antibiotic treatment (13 out of 16) belonged to the infection group. Figure 1 presents the relative abundance of the ten most abundant bacterial genera in each patient and sample. In 35 of the samples (78%), bacterial species other than normal oral flora were identified in the conventional culture. All culture findings were identified in the corresponding microbiome analyses, and in 20 samples (57% of the culture positive samples) the bacterium found in cultures was the dominating species in the microbiome. Of these 20 samples, 17 (85%) were collected during ongoing airway infection, compared to 9 of the 15 samples (60%) without a dominating culture finding in the microbiome (*p* = 0.13).

**Table 1:** Patient characteristics

Total number of patients; n	22
Age; median (range)	57 (24–65)
Male gender; n (%)	13 (59)
Type of lung Tx; n (%)	
Single Double	4 (18) 18 (82)
Underlying diagnosis; n (%)	
Cystic fibrosis Fibrosis Emphysema COPD PAH BOS Sarcoidosis GVH	6 (27) 5 (23) 3 (14) 3 (14) 2 (9) 1 (5) 1 (5) 1 (5)

*COPD* Chronic Obstructive Pulmonary Disease

*PAH* Pulmonary Arterial Hypertension

*BOS* Bronchiolitis Obliterans Syndrome

*GVH* Graft versus host disease

**Table 2:** BALF sample characteristics

Number of BALF samples	46
Samples/patient; median (range)	2 (1–4)
Sample collection during; n (%)	
No infection Infection Antibiotic treatment	15 (33) 31 (67) 16 (35)
Bacterial growth in conventional cultures; n (%)*	
* Pseudomonas aeruginosa* * Escherichia coli* * Stenotrophomonas maltophilia* * Burkholderia spp* * Staphylococcus aureus* Other bacteria Negative culture	9 (20) 8 (18) 5 (11) 4 (9) 4 (9) 9 (20) 11 (24)

*Several bacterial species may be found in one culture, and the total number of findings can therefore exceed the number of samples

### Species diversity and microbiome composition is altered during infection and inflammation

**Fig. 2:**
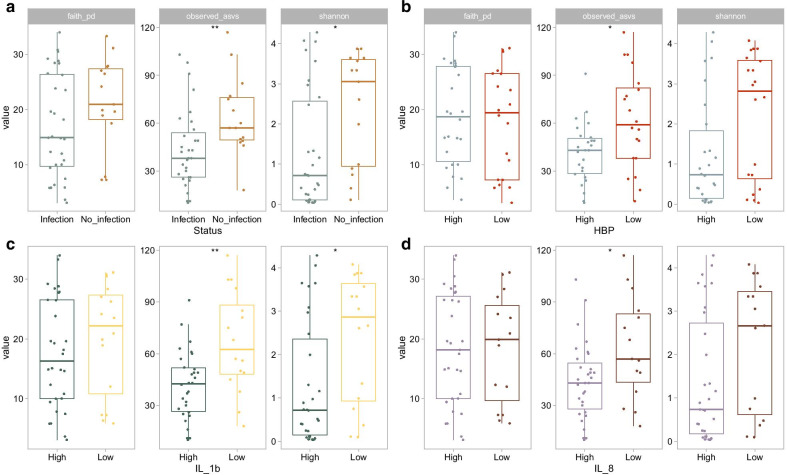
Alpha-diversity of BALF samples. The microbiome composition of each sample was assessed based on phylogenetic diversity (faith; left panels), species richness (number of observed ASVs; middle panels) and ASV diversity (shannon index; right panels). Samples were compared based on clinical signs of infection at the time of sampling (**a**) and high versus low BALF-levels of the inflammatory markers HBP (**b**), IL-1β (C) or IL-8 (D). *p < 0.05, **p < 0.01

Alpha diversity metrics within each sample was assessed by calculating phylogenetic diversity (faith), species richness (number of observed ASVs) and species diversity (shannon index). The microbiomes of samples collected during ongoing infection had a significantly lower number of observed ASVs and a lower shannon index, indicating a lower species diversity, compared to samples from non-infected patients (Fig. 2a). No difference in alpha-diversity was found between samples with or without on-going antibiotic treatment [Additional file 1: Figure S1]. In a sub-analysis on non-antibiotic samples only, the same trend with lower species richness in samples collected during infection was seen [Additional file 2: Figure S2]. Next, samples with high and low BALF levels of the inflammatory biomarkers HBP, IL-1β and IL-8 were compared. HBP concentrations above 150 ng/mL in BALF were regarded as high, whereas the cut-off levels for IL-1β and IL-8 were 10 and 1 ng/mL, respectively [8]. As seen in Fig. 2b–d, the number of observed ASVs was significantly higher in samples with low levels of the biomarkers. Samples with low concentrations of IL-1β also had a higher Shannon index, suggesting a higher diversity within this group of samples.

**Fig. 3:**
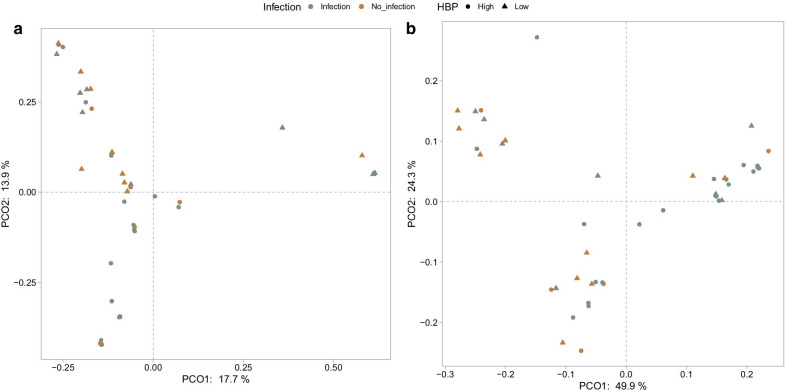
Beta-diversity of the BALF samples. Principal coordinate analysis (PCoA) plots demonstrating the distance in the microbiome composition between samples classified as infection (grey) versus no infection (brown), and between samples with high (circles) versus low (triangles) levels of HBP. **a** Shows differences in abundance calculated with Bray–Curtis distance, where PEMANOVA analyses demonstrated a significant difference in the distance between both infection vs no infection samples (*p* < 0.05) and high vs low levels of HBP (*p* < 0.05). **b** Shows the phylogenetic distance between samples calculated with weighted UniFrac analyses, demonstrating a significant difference between infection vs no infection samples (*p* < 0.05) as well as between high vs low levels of HBP (*p* < 0.05)

Differences in the microbiome composition between the study samples were compared using Bray–Curtis distance and weighted or unweighted UniFrac. A significant difference in the microbiome composition was detected when samples collected during infection was compared to non-infection samples using Bray–Curtis (*p* < 0.05) (Fig. 3a) and weighted UniFrac (*p* < 0.05) (Fig. 3b). When samples with high and low levels of the biomarkers were compared, a significant difference was found for HBP using Bray–Curtis (*p* < 0.05) (Fig. 3a) and weighted UniFrac analyses (*p* < 0.05) (Fig. 3b), and for IL-1β in the weighted UniFrac analyses (*p* < 0.05)[ Additional file 3: Figure S3]. No differences in the microbiome composition was found in samples with high versus low IL-8 concentrations.

Patients with on-going antibiotic treatment at the time of sampling had a significantly different microbiome composition compared to patients without antibiotics (p < 0.01 in the Bray–Curtis analyses and *p* < 0.01 with Weighted UniFrac)[ Additional file 4: Figure S4], and patients with cystic fibrosis (CF) had a significantly different microbiome compared to patients with other underlying diseases (*p* < 0.001, *p* < 0.05 and *p* < 0.05 using Bray–Curtis, Weighted UniFrac and Unweighted UniFrac, respectively) [Additional file 4: Figure S4].

### Enrichment of species during infection, inflammation and antibiotic treatment

**Fig. 4:**
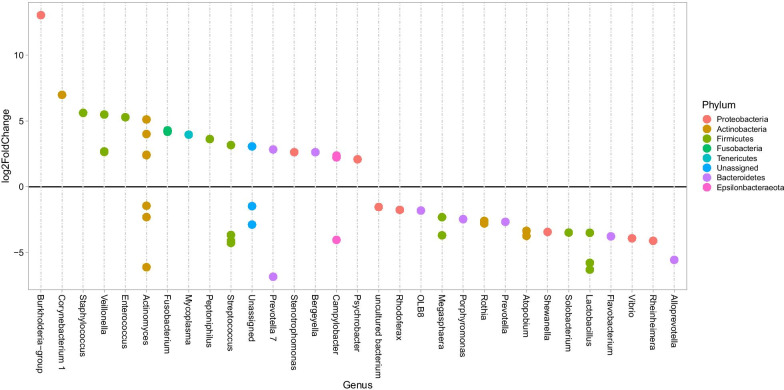
Enrichment of microbes during infection. Differential abundance analysis of samples graded as infection versus no infection was used to identify enriched organisms during infection. The figure shows ASVs with adjusted *p*-values < 0.01. Each circle represents an ASV, and all ASVs with a log2FoldChange above zero are significantly enriched during infection. The different colours represent different phyla

Enrichment analyses identified several genera that were more commonly found during episodes of infection (Fig. 4). The most enriched genus was *Burkholderia*-group, represented by *Burkholderia multivorans* on the species level. *Corynebacterium* and *Staphylococcus*, represented by *S. aureus* on the species level, were also found among the enriched species during infection. Among the less abundant genera during infection, several anaerobes believed to be part of the normal flora were identified, such as *Lactobacillus*, *Prevotella*, *Porphyromonas* and *Rothia* (Fig. 4). Some genera, such as *Streptococcus*, contained both enriched and less abundant species during infection. Analyses on the species level for the *Streptococcus* genus showed that an uncultured species was enriched during infection whereas *S. equinus* was less abundant [see Additional file 9: Table S3]. When samples with high or low inflammatory biomarkers were compared, *Burkholderia*-group was again the most enriched genus in samples with high levels of HBP, IL-1β or IL-8. *S. aureus* and *Corynebacterium* were also enriched in all three groups, whereas anaerobes and normal oral flora were less abundant [see Additional file 10: Table S4].

In samples collected during on-going antibiotic treatment, *Enterococcus*, *Mycoplasma*, *Staphylococcus* and *Pseudomonas* were among the most enriched species [Additional file 5: Figure S5]. Anaerobes and oral flora, including *Lactobacillus*, *Streptococcus*, *Prevotella* and *Haemophilus*, were again less abundant.

Given that *Burkholderia*-group was the most enriched species during infection and inflammation, and that all study samples dominated by *Burkholderia*-group were from the same patient, we repeated all analyses without this specific patient. The results of the alpha-diversity analyses were similar to the results for the whole group, with significantly lower numbers of observed ASVs during infection and inflammation. When comparing the microbiome composition, the distance between infection and non-infection samples was no longer significant in the Bray–Curtis analyses (*p* = 0.06), whereas the difference in microbiome composition between samples collected during antibiotic treatment or not was still highly significant (*p* < 0.01). As expected, *Burkholderia*-group was no longer enriched during infection or in samples with high levels of the inflammatory markers [Additional file 6: Figure S6].

## Discussion

In this prospective study on LTx patients, the microbiome of patients with airway infection was characterized by loss of bacterial diversity. Similar results were obtained both when samples were classified based on clinical signs of infection in the patient, and on the degree of inflammation in the sample. These results are in agreement with previous studies that also reported loss of microbiome diversity in LTx recipients during infection [22, 23]. Moreover, the microbiome composition was significantly different in samples collected during infection versus non-infection, and in samples with high versus low levels of the inflammatory biomarkers HBP and IL-1β. *Burkholderia*, *Corynebacterium* and *Staphylococcus aureus* were significantly enriched during infection and inflammation, whereas anaerobes and normal oropharyngeal flora were underrepresented. Although we can’t prove that the enriched species are causative of infection, both *Burkholderia* and *S. aureus* are recognized as important pathogens in LTx recipients [3, 24–26]. *Corynebacterium* has been reported to be enriched in patients with chronic rhinosinusitis [27], but has not been described in LTx recipients before.

The lung microbiome of healthy individuals is characterized by a low bacterial load and a high species diversity, with the most common genera being *Prevotella*, *Streptococcus*, *Veillonella*, *Neisseria*, *Haemophilus*, and *Fusobacterium* [28, 29]. In this study, *Prevotella* was found among the under-represented species during infection and inflammation. In agreement with our findings, a *Prevotella*-dominated microbiome has been reported to be underrepresented during inflammation in LTx patients [30]. Moreover, *Prevotella* has been shown to be less pro-inflammatory in vivo [30] and to induce a weaker cytokine response in murine models of airway infections compared to pathogens associated with disease in Chronic Obstructive Pulmonary Disease (COPD) [31]. On the other hand, *Veillonella* and *Fusobacterium*, that are part of the normal lung microbiome, were enriched during inflammation and infection. However, the microbiome of the transplanted lung is most likely different from that of the healthy lung due to immunosuppressive therapy, altered immune defences and high use of antibiotics. For example, it has been shown that LTx recipients have a higher bacterial burden in the lower airways compared to healthy controls [32]. It is possible that less virulent bacteria may cause infection in this group of patients.

Antibiotic treatment at the time of sampling did not affect species richness and abundance of the study samples, which is in agreement with previous reports [23]. However, antibiotic treatment was one of the parameters that affected the beta-diversity the most in this study. These differences in microbiome composition were more significant than when samples were compared based on clinical signs of infection or level of inflammation. Importantly, many of the species that were enriched during antibiotic treatment, for example *Enterococcus*, *Mycoplasma* and *Pseudomonas*, are resistant to commonly used antibiotics. Therefore, we can’t know whether these species are more virulent and cause infection that need antibiotic treatment, or if they are enriched during antibiotic treatment because of their resistance to antimicrobials.

All bacterial species found in BALF cultures in our sample cohort were also identified in the corresponding bacterial microbiome, although not always the dominating species. Surprisingly, none of the three most common species found in BAL cultures, including *P. aeruginosa*, were enriched during infection or inflammation (Table 2 and Fig. 3). *P. aeruginosa* has previously been reported as an important pathogen in culture-based studies on LTx recipients with pneumonia [3, 5, 33], and it was found to be more abundant in the microbiome of LTx patients with signs of acute infection in a study by Dickson et al. [23]. Many LTx recipients, and in particular patients with CF, are chronically colonized with *Pseudomonas* in the lung, and findings of *P. aeruginosa* in cultures could within the same patient represent either chronic colonization or acute infection at different time points. In line with this hypothesis, a previous microbiome study on longitudinal sputum samples from COPD patients suggested that the subjects had individual microbiomes that were distinct from each other, and that exacerbations were likely to result from changes in the relative abundance of pre-existing bacteria rather than removal or appearance of existing species [34]. This could also fit well with a proposed conceptual model of airway infection, where the development of pneumonia results from a disruption in the complex homeostasis of the lung microbiome [35].

An important limitation of this study is the small number of patients, and the results must therefore be interpreted with caution. In addition, the study participants contributed with different numbers of samples to the study. For example, all samples with *Burkholderia*-group were from the same patient. To address this possible bias, we did analyses both with and without this specific patient. Most results were still comparable, with the exception that the differences in microbiome composition during infection and non-infection was just above the limit for significance in the Bray–Curtis analyses (*p* = 0.06). Even so, the enrichment analyses gave similar results, indicating that this patient did not bias the results other than for *Burkholderia*-group. Another difficulty is that our cohort included patients with different underlying diseases. This could affect the results, as the microbiome may vary depending on the underlying diagnosis. For example, we could show that CF-patients had a significantly different microbiome composition compared to other patients [Additional file 4: Figure S4]. This is probably explained by the fact that CF patients are colonized with bacteria in both the upper and lower airways, and that the sinuses are an important reservoir for bacteria that can re-infect the graft [36, 37].

In conclusion, the airway microbiome of LTx recipients is characterized by loss of bacterial diversity and altered microbiome composition during infection. The most common species in BALF cultures were not found among the enriched species in the microbiome during infection. Taken together, the data suggests that airway infections are associated with a disturbed balance of the microbiome, and emphasizes the difficulty in interpreting BALF culture results from these patients.

## Supplementary Information

**Additional file 1: Figure S1.** Alpha-diversity in samples collected during antibiotic treatment. The microbiome composition of each sample was assessed based on phylogenetic diversity (faith; left panels), species richness (number of observed ASVs; middle panels) and ASV diversity richness combined with abundance (shannon index; right panels). No significance was found in any of the three metric comparisons.

**Additional file 2: Figure S2.** Comparison of alpha-diversity metrics between infection and non-infection samples after exclusion of samples collected during antibiotic treatment. Phylogenetic diversity (faith; left panels), species richness (number of observed ASVs; middle panels) and ASV diversity richness combined with abundance (shannon index; right panels) were compared between infection and non-infection samples. Only samples without antibiotic treatment at the time of sampling were included in the analyses (n = 30). No significance was found in any of the three metric comparisons.

**Additional file 3: Figure S3.** Comparison of microbiome beta-diversity in relation to IL-1β concentrations. PCoA plot of the distance in the microbiome composition between samples with high (grey) versus low (yellow) levels of IL-1β. The distances are calculated with weighted UniFrac and are significantly different (*p* ≤ 0.05).

**Additional file 4: Figure S4.** Beta-diversity in relation to antibiotic treatment and underlying diagnosis. PCoA plots of the distances in the microbiome composition between samples collected during ongoing antibiotic treatment (blue) or no antibiotic treatment (red), and between samples from patients with cystic fibrosis (CF; circles) compared to other underlying diagnoses (triangles). **a** Shows Bray–Curtis distances, where significant differences in the microbiome composition were found between samples collected during antibiotic treatment compared to no antibiotic treatment (*p* < 0.01), and between samples from patients with CF compared to other underlying conditions (*p* < 0.001). **b** Shows differences calculated with weighted UniFrac. Significant differences were found between samples with or without ongoing antibiotic treatment at the time of sampling (*p* < 0.01) and between samples from CF-patients versus non-CF patients (*p* < 0.05).

**Additional file 5: Figure S5.** Enrichment analyses of species in samples collected during antibiotic treatment. Each dot represents an ASV, and ASVs with a log2FoldChange above zero are enriched during antibiotic treatment, whereas ASVs below zero are less abundant. Only ASVs with adjusted *p*-values < 0.01 are plotted in the figure. The different colours represent different bacterial phyla.

**Additional file 6: Figure S6.** Enrichment analyses of bacterial species in samples graded as infection. Five BALF samples with dominance of Burkholderia-group in the microbiome, all from the same patient, were excluded from this analysis in order to assess the possible bias of the results due to these samples. Each dot represents an individual ASV and only ASVs with adjusted *p*-values < 0.01 are plotted in the figure. ASVs with a log2FoldChange above zero are enriched during antibiotic treatment, whereas ASVs below zero are less abundant. The different colours represent different bacterial phyla.

**Additional file 7: Table S1.** Grading of infection.

**Additional file 8: Table S2.** Patient and sample characteristics.

**Additional file 9: Table S3.** Enrichment analysis for samples collected during infection.

**Additional file 10: Table S4.** Enrichment analysis for the three inflammatory biomarkers.

## Data Availability

The datasets analysed during the current study are available from the corresponding author on reasonable request.

## Abbreviations

ASV: Amplicon sequence variants
BAL: Bronchoalveolar lavage
BALF: Bronchoalveolar lavage fluid
BOS: Bronchiolitis Obliterans Syndrome
CF: Cystic fibrosis
COPD: Chronic Obstructive Pulmonary Disease
GVH: Graft versus host disease
HBP: Heparin-binding protein
LTx: Lung transplant
PAH: Pulmonary Arterial Hypertension
