# Supplementary figures and images for: Divergent airway microbiomes in lung transplant recipients with or without pulmonary infection
Source: Respir Res. 2021 Apr 23;22:118. doi: 10.1186/s12931-021-01724-w (PMC8063417; doi:10.1186/s12931-021-01724-w)

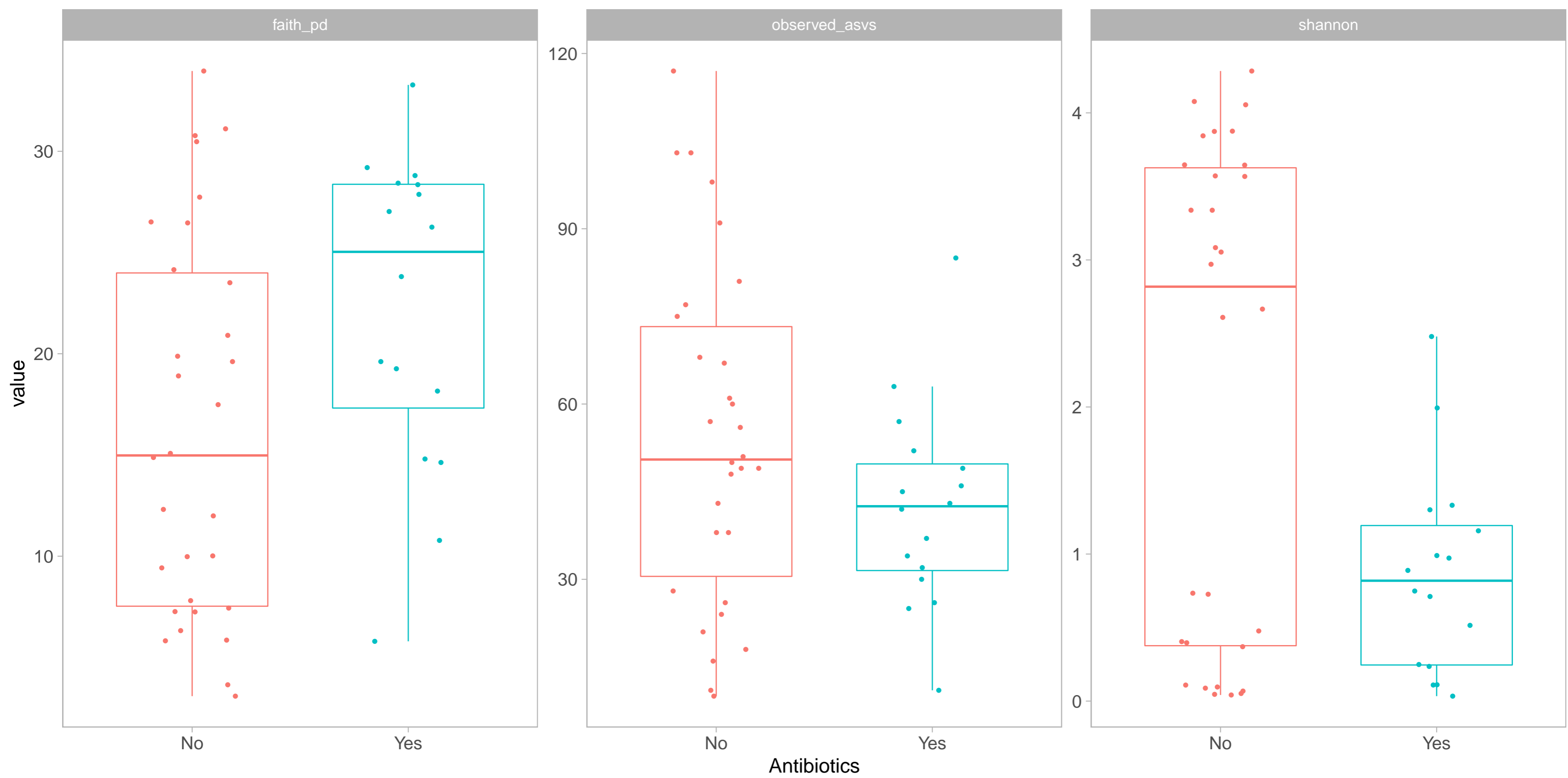

Supplement: Supplementary file 1 — Additional file 1: Figure S1. Alpha-diversity in samples collected during antibiotic treatment. The microbiome composition of each sample was assessed based on phylogenetic diversity (faith; left panels), species richness (number of observed ASVs; middle panels) and ASV diversity richness combined with abundance (shannon index; right panels). No significance was found in any of the three metric comparisons. [file 12931_2021_1724_MOESM1_ESM.pdf]

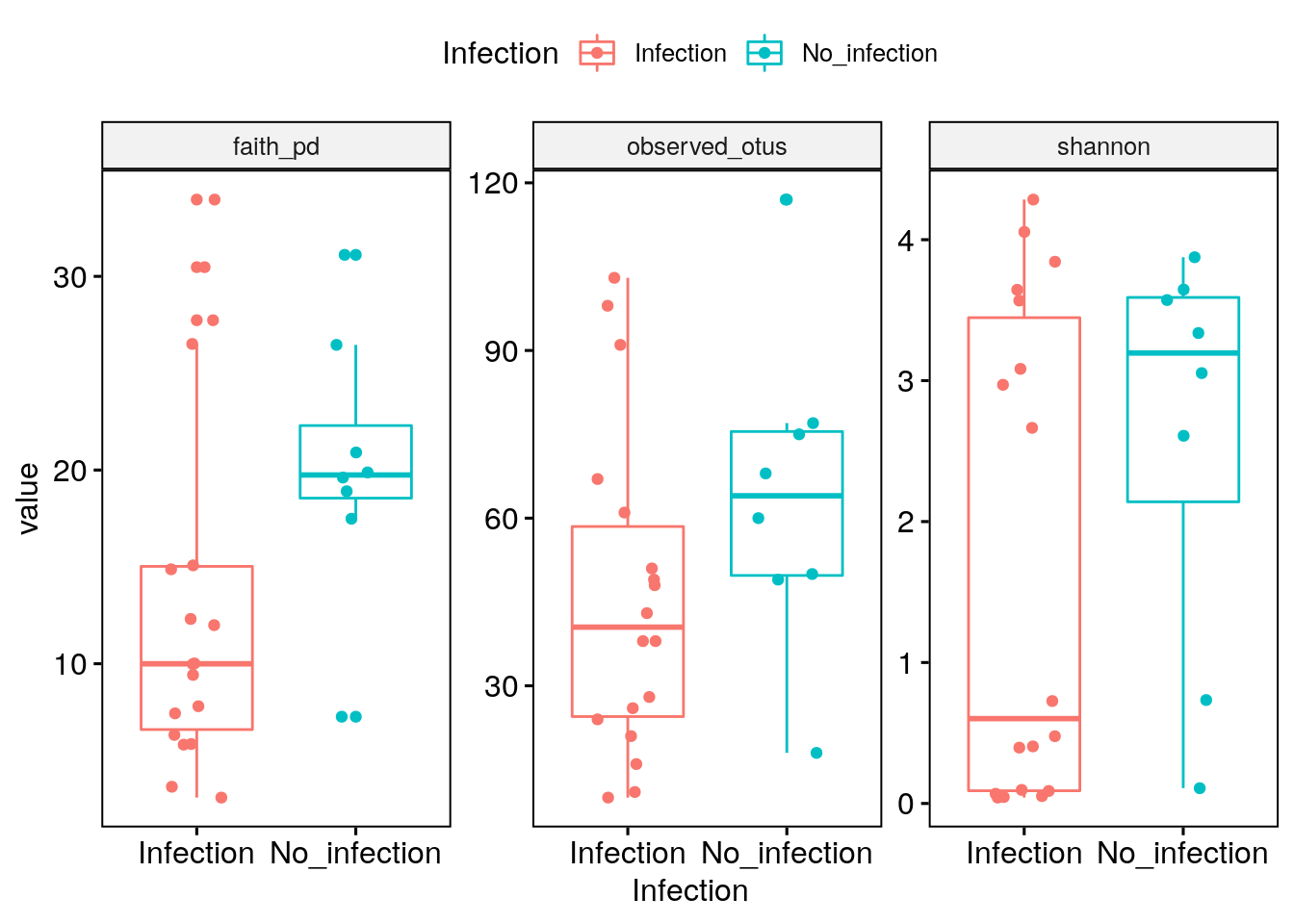

Supplement: Supplementary file 2 — Additional file 2: Figure S2. Comparison of alpha-diversity metrics between infection and non-infection samples after exclusion of samples collected during antibiotic treatment. Phylogenetic diversity (faith; left panels), species richness (number of observed ASVs; middle panels) and ASV diversity richness combined with abundance (shannon index; right panels) were compared between infection and non-infection samples. Only samples without antibiotic treatment at the time of sampling were included in the analyses (n = 30). No significance was found in any of the three metric comparisons. [file 12931_2021_1724_MOESM2_ESM.png]

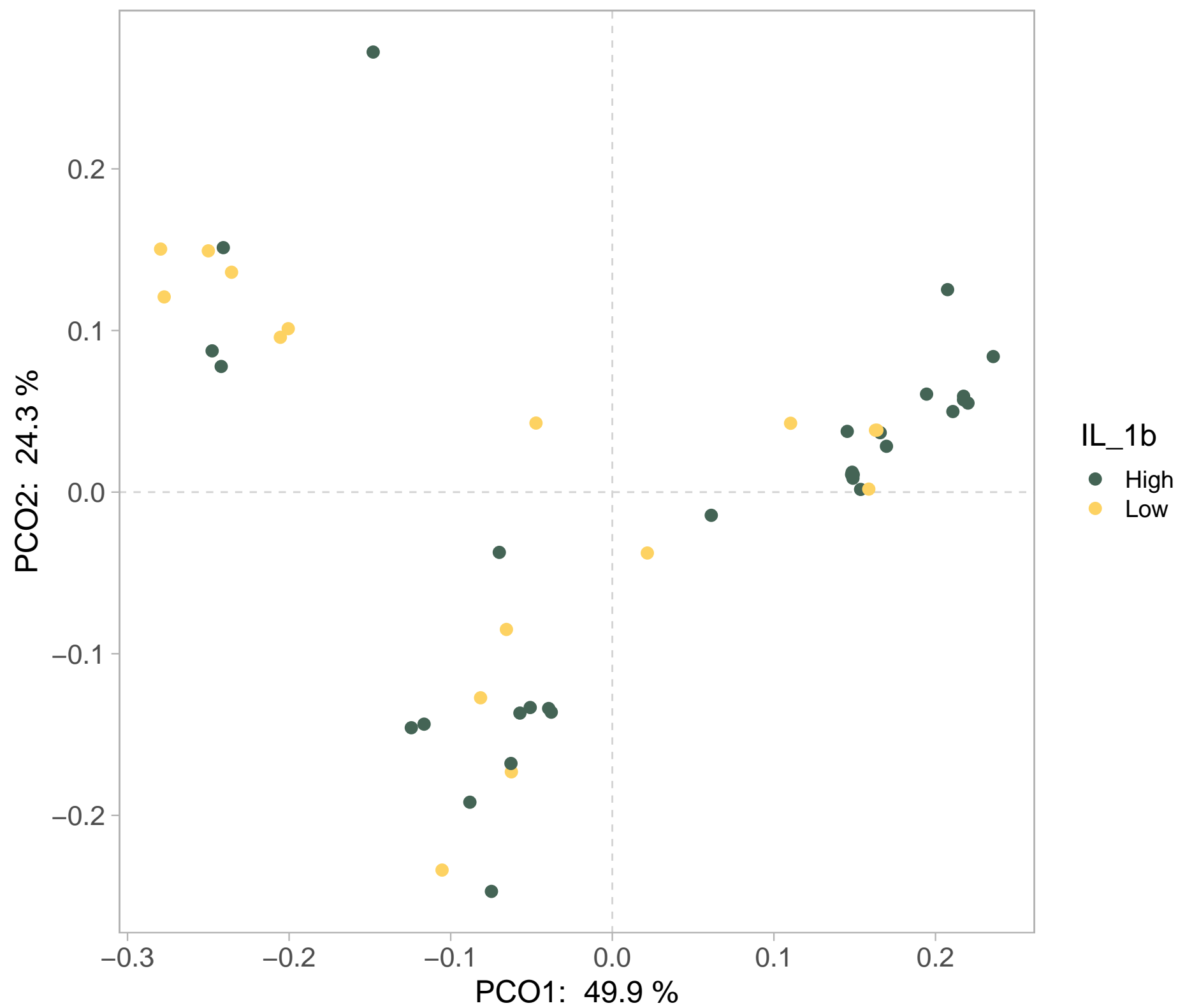

Supplement: Supplementary file 3 — Additional file 3: Figure S3. Comparison of microbiome beta-diversity in relation to IL-1β concentrations. PCoA plot of the distance in the microbiome composition between samples with high (grey) versus low (yellow) levels of IL-1β. The distances are calculated with weighted UniFrac and are significantly different (p ≤ 0.05). [file 12931_2021_1724_MOESM3_ESM.pdf]

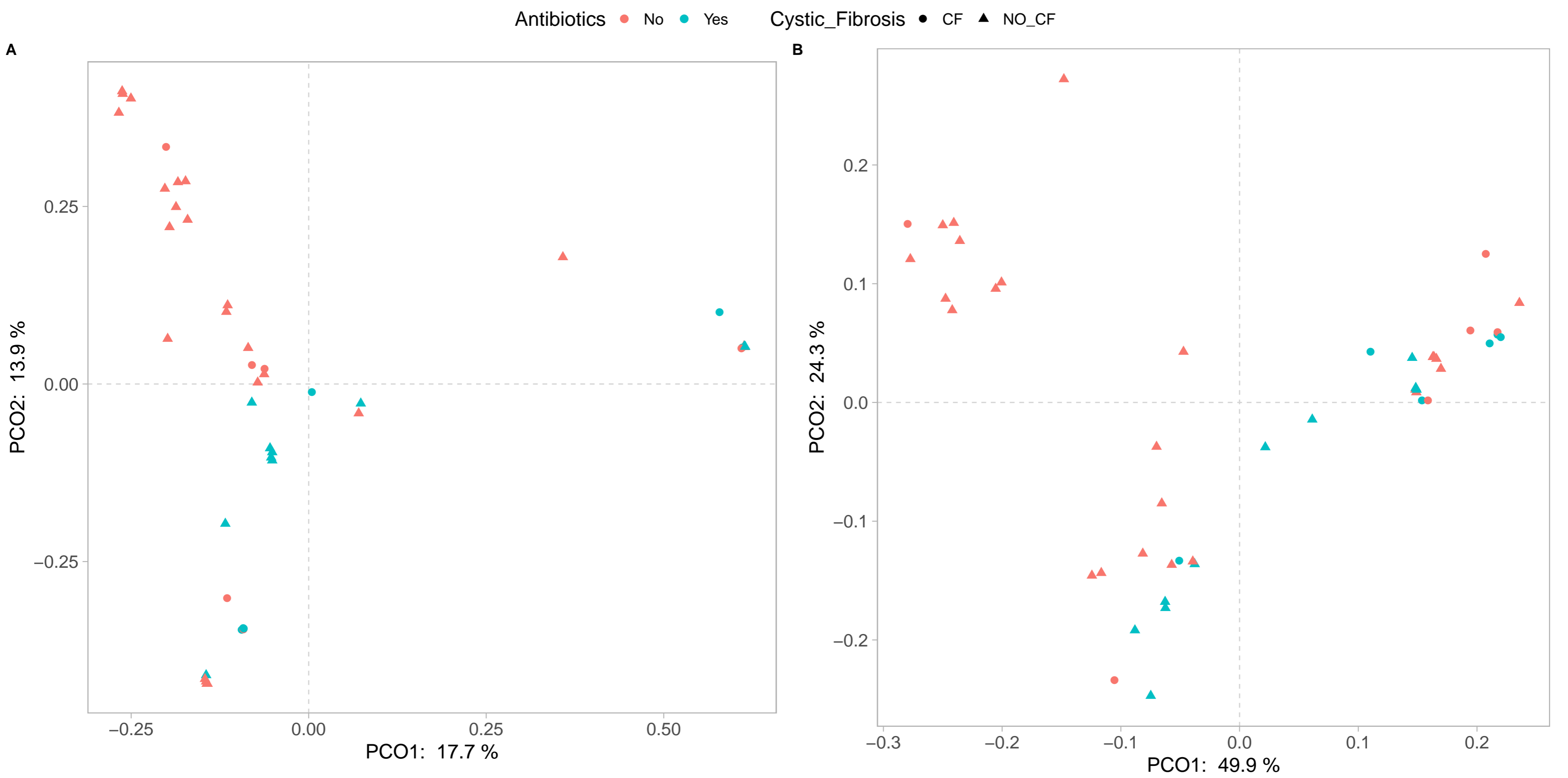

Supplement: Supplementary file 4 — Additional file 4: Figure S4. Beta-diversity in relation to antibiotic treatment and underlying diagnosis. PCoA plots of the distances in the microbiome composition between samples collected during ongoing antibiotic treatment (blue) or no antibiotic treatment (red), and between samples from patients with cystic fibrosis (CF; circles) compared to other underlying diagnoses (triangles). a Shows Bray–Curtis distances, where significant differences in the microbiome composition were found between samples collected during antibiotic treatment compared to no antibiotic treatment (p < 0.01), and between samples from patients with CF compared to other underlying conditions (p < 0.001). b Shows differences calculated with weighted UniFrac. Significant differences were found between samples with or without ongoing antibiotic treatment at the time of sampling (p < 0.01) and between samples from CF-patients versus non-CF patients (p < 0.05). [file 12931_2021_1724_MOESM4_ESM.pdf]

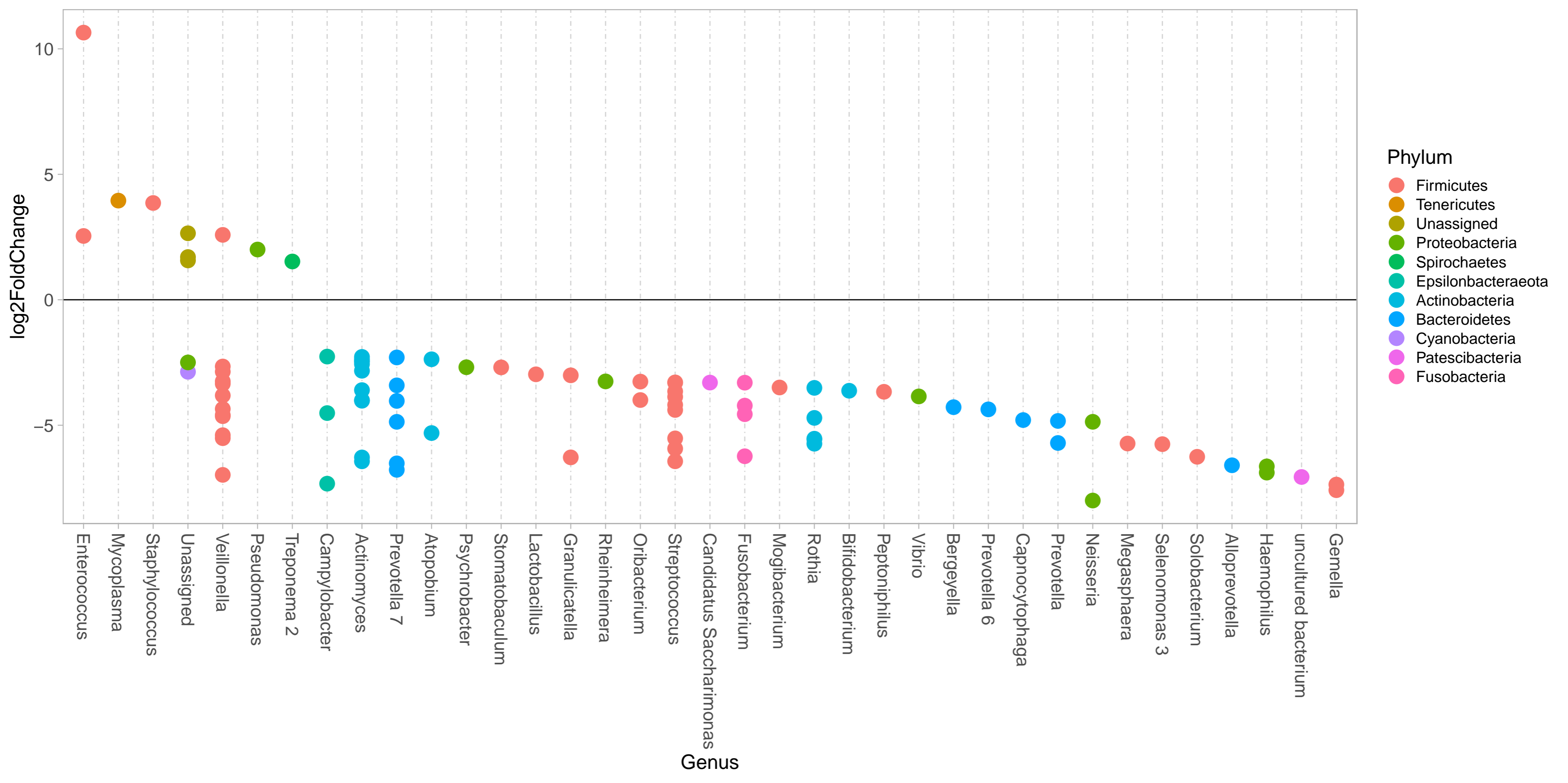

Supplement: Supplementary file 5 — Additional file 5: Figure S5. Enrichment analyses of species in samples collected during antibiotic treatment. Each dot represents an ASV, and ASVs with a log2FoldChange above zero are enriched during antibiotic treatment, whereas ASVs below zero are less abundant. Only ASVs with adjusted p-values < 0.01 are plotted in the figure. The different colours represent different bacterial phyla. [file 12931_2021_1724_MOESM5_ESM.pdf]

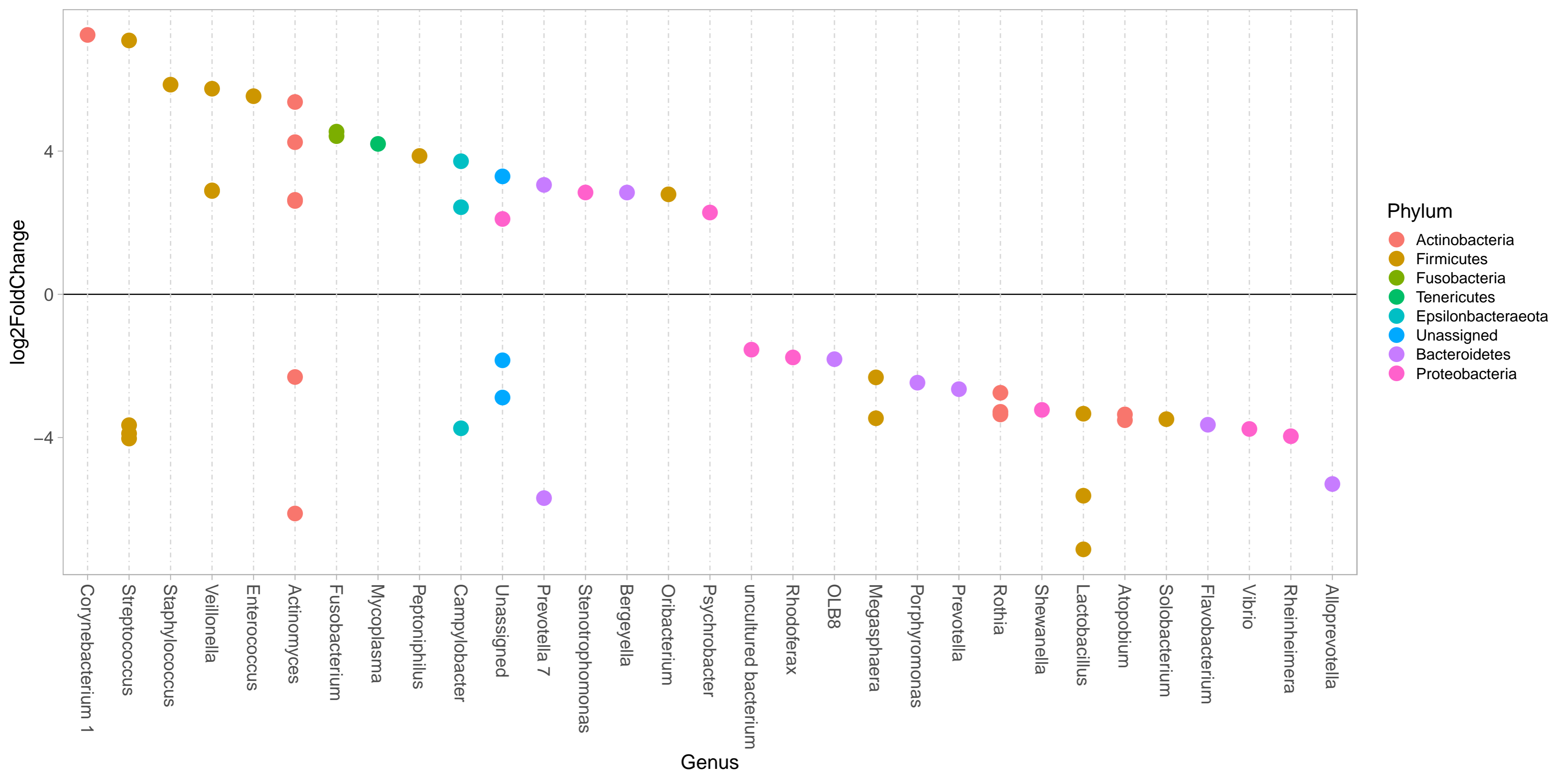

Supplement: Supplementary file 6 — Additional file 6: Figure S6. Enrichment analyses of bacterial species in samples graded as infection. Five BALF samples with dominance of Burkholderia-group in the microbiome, all from the same patient, were excluded from this analysis in order to assess the possible bias of the results due to these samples. Each dot represents an individual ASV and only ASVs with adjusted p-values < 0.01 are plotted in the figure. ASVs with a log2FoldChange above zero are enriched during antibiotic treatment, whereas ASVs below zero are less abundant. The different colours represent different bacterial phyla. [file 12931_2021_1724_MOESM6_ESM.pdf]
