# Supplementary material for: Divergent airway microbiomes in lung transplant recipients with or without pulmonary infection
Source: Respir Res. 2021 Apr 23;22:118. doi: 10.1186/s12931-021-01724-w (PMC8063417; doi:10.1186/s12931-021-01724-w)
Supplement: Supplementary file 7 — Additional file 7: Table S1. Grading of infection. [file 12931_2021_1724_MOESM7_ESM.docx]

**Additional Table 1.** Grading of infection

|  | **A. Radiology** | **B. Bronchoscopy** | **C. Clinical criteria** | **D. Microbiology** |
| --- | --- | --- | --- | --- |
|  | New or increasing radiographic changes on chest X-ray or CT scan | One or more of the following endobronchial abnormalities:   - Inflamed endobronchial mucosa - Endobronchial lesion (white/yellow) with/without necrotic changes - Purulent secretion - Inflammatory cells in cytology and/or PAD | One or more of the following conditions:   - New or increased cough, dyspnoea, increased sputa - Fever >38°C - Worsened gas exchange - White blood cell count>15 | Bacterial growth in BALF culture (other than normal oropharyngeal flora) |
| Definite infection | A, B, C and D | | | |
| Probable infection | A and/or B | | C and/or D | |
| Possible infection | One criterium (A, B, C or D) | | | |
| No infection | No | No | No | No |

BALF = Bronchoalveolar lavage fluid

CT = computed tomography

PAD = pathological anatomical diagnosis
