# Supplementary material for: Divergent airway microbiomes in lung transplant recipients with or without pulmonary infection
Source: Respir Res. 2021 Apr 23;22:118. doi: 10.1186/s12931-021-01724-w (PMC8063417; doi:10.1186/s12931-021-01724-w)
Supplement: Supplementary file 8 — Additional file 8: Table S2. Patient and sample characteristics. [file 12931_2021_1724_MOESM8_ESM.pdf]

Additional table 2. Patient and sample characteristics

Patient ID	Pre-transplant diagnosis	Immunosuppression	sample ID	Months after Tx	Bacterial growth in BALF culture	Infection status (0=no infection)
P1	COPD	Ciclosporin, Azathioprin	120	3,2	CoNS, *Stenotrophomonas maltophilia*	Infection
P1			195	6,4	oropharyngeal flora	0
P2	Fibrosis	Ciclosporin, Azathioprin	37	5,3	*Escherichia coli ESBL*	Infection
P3	Emphysema	Tacromilus, Mycophenolate	126	2,3	CoNS	0
P3			139	2,5	*Enterococcus faecium*	Infection
P3			179	3,9	CoNS	0
P3			268	9,5	*Pseudomonas aeruginosa*	Infection
P4	CF	Ciclosporin, Azathioprin	114	2,8	0	0
P4			185	6,1	0	0
P5	Fibrosis	Tacromilus, Mycophenolate	207	3,1	*Staphylococcus aureus*	Infection
P6	COPD	Ciclosporin, Azathioprin	111	3,7	*Staphylococcus aureus*	Infection
P6			210	7,9	*Streptococcus pneumoniae*	Infection
P7	PAH	Tacromilus, Mycophenolate	181	1,2	0	Infection
P7			199	2,5	oropharyngeal flora	0
P8	Fibrosis	Tacromilus, Mycophenolate	192	2,7	*Stenotrophomonas maltophilia*	Infection
P8			221	3,6	*Escherichia coli*	Infection
P8			223	3,9	*Escherichia coli*	Infection
P8			238	4,8	*Stenotrophomonas maltophilia*	Infection
P8			347	11,1	*Stenotrophomonas maltophilia*	0
P9	CF	Tacromilus	391	6	0	0
P10	Emphysema	Tacromilus	256	0,9	0	0
P10			259	1,1	0	Infection
P10			299	2,9	CoNS	0
P11	CF	Ciclosporin, Azathioprin	325	2,8	*Staphylococcus aureus, Pseudomonas aeruginosa*	Infection
P12	CF	Ciclosporin, Azathioprin	240	0,8	Oropharyngeal flora	Infection
P12			270	3,1	*Burkholderia cepacia complex*	Infection
P12			294	4,3	*Burkholderia cepacia complex*	Infection
P12			328	6,4	*Burkholderia cepacia complex*	Infection

P12			376	8,2	*Burkholderia cepacia complex*	Infection
P13	GVH	Tacromilus, Mycophenolate	318	2,5	*Pseudomonas aeruginosa*	Infection
P13			370	4,7	*Escherichia coli, Pseudomonas aeruginosa*	0
P14	COPD	Tacrolimus	330	1,3	*Enterococcus faecium, Staphylococcus aureus*	Infection
P14			458	10,3	*Pseudomonas aeruginosa*	Infection
P15	Sarcoidosis	Tacromilus, Mycophenolate	404	3,2	Oropharyngeal flora	0
P16	Fibrosis	Ciclosporin, Mycophenolate	385	4,4	*Pseudomonas aeruginosa*	Infection
P16			409	6	*Enterobacter cloacae*	0
P17	CF	Tacromilus, Mycophenolate	423	0,2	*Pseudomonas aeruginosa, Stenotrophomonas maltophilia*	Infection
P17			510	9,5	0	Infection
P18	Fibrosis	Tacrolimus	429	3	*Escherichia coli*	Infection
P18			470	6,1	*Eschericha coli*	Infection
P19	PAH	Tacromilus, Mycophenolate	448	2,7	*Escherichia coli*	0
P19			487	6	*Escherichia coli*	Infection
P20	Emphysema	Ciclosporin, Azathioprin	460	2	*Pseudomonas aeruginosa*	Infection
P20			502	5,2	*Pseudomonas aeruginosa*	Infection
P21	CF	Tacromilus, Mycophenolate	493	3	*Achromobacter xylosoxidans, Staphylococcus simulans*	Infection
P22	BOS	Tacromilus, Mycophenolate	466	3	*Serratia marsecens,* oropharyngeal flora	0

BOS = Bronchiolois Obliterans Syndrome
CF = Cystic fibrosis
CoNS = Coagulase negative Staphylococcus
COPD = Chronic Obstructive Pulmonary Disease
GVH = Graft versus host disease
PAH = Pulmonary Arterial Hypertension
