# Supplementary material for: Divergent airway microbiomes in lung transplant recipients with or without pulmonary infection
Source: Respir Res. 2021 Apr 23;22:118. doi: 10.1186/s12931-021-01724-w (PMC8063417; doi:10.1186/s12931-021-01724-w)
Supplement: Supplementary file 9 — Additional file 9: Table S3. Enrichment analysis for samples collected during infection. [file 12931_2021_1724_MOESM9_ESM.pdf]

Additional table 3: Enrichment analysis for samples collected during infection.
If the log2FoldChange is above ZERO, meaning that the ASVs were more abundant during infection, and below ZERO is more abundant in non-infection

ASV_ID	Phylum	Genus	Species	log2FoldChange	padj
0b18ca83ef493094b8d6965fdd13d3ad	Actinobacteria	Atopobium	uncultured bacterium	-3,741773221	5,90168E-05
0bc2ff6e68ca5e252d55825929ba4201	Epsilonbacteraeota	Campylobacter	Unassigned	2,371014876	0,006403302
0f610832752125a87bb4da98d00e03c6	Fusobacteria	Fusobacterium	uncultured organism	4,293525622	6,54716E-05
11b41270aa0f2ee0cb25acad36d397e9	Proteobacteria	Shewanella	Unassigned	-3,439050959	5,17322E-05
1408b62055d449b2abb904abee855875	Firmicutes	Veillonella	Unassigned	5,494528408	6,32774E-08
1910dcf6315135f7947c373ec31f82f6	Firmicutes	Streptococcus	uncultured bacterium	3,166903258	0,000355879
22f1c5d4e53e8c961dd73e244503e7b7	Firmicutes	Streptococcus	uncultured bacterium	-4,27898603	0,000113907
2679ce9192f4b9a7fac20960748935b8	Proteobacteria	Rhodoferax	uncultured bacterium	-1,761945863	0,000782086
28b8b8712009fcc7f5fce3d86e1bf647	Bacteroidetes	Flavobacterium	uncultured bacterium	-3,77651285	2,46835E-07
2b3e246dc92bce326698cc60a8608a2f	Bacteroidetes	Alloprevotella	uncultured bacterium	-5,568177261	2,51974E-10
2e5242ae8c79e05740b498c1233602ac	Unassigned	Unassigned	Unassigned	3,068111619	0,000782086
3041019246205852cbbcce91bef2fef8	Firmicutes	Megasphaera	Unassigned	-2,31741676	0,000154329
33f1205eacafe6210d21e955135800d6	Firmicutes	Streptococcus	Streptococcus equinus	-4,115650726	3,09737E-06
34cc9d0ff9bc451e9da9156b90906df6	Bacteroidetes	Porphyromonas	uncultured bacterium	-2,466718724	8,25362E-05
38b502eac93359dc19677061e0b4e889	Actinobacteria	Rothia	uncultured bacterium	-2,796929816	9,47109E-06
4fa7999ddeac300066f4e86af2e94fc6	Bacteroidetes	Bergeyella	uncultured bacterium	2,62784441	0,00239054
5818cd73593b458018d6941c486dcbe3	Firmicutes	Solobacterium	Unassigned	-3,489263348	6,15672E-07
58be7fff0121d20b6c908108fb0f5e37	Epsilonbacteraeota	Campylobacter	Unassigned	-4,046684412	2,04621E-05
64f64f158cf671861b380e95591c1d98	Firmicutes	Staphylococcus	Staphylococcus aureus	5,620315775	1,29847E-06
666c842b3a3a5756feed9bb2856d76fc	Proteobacteria	Psychrobacter	Unassigned	2,088418901	0,009128321
6c247835d06811be636d8bffcf005b3d	Actinobacteria	Actinomyces	Unassigned	-6,122029971	5,27356E-18
6ddebcc029eb24f1ca349b7345747970	Bacteroidetes	Prevotella 7	uncultured bacterium	2,834197388	0,001192319
6de9ef7fce39ce78ddf58c10d61f8341	Epsilonbacteraeota	Campylobacter	uncultured bacterium	2,231648045	0,008286215
6fcbf58d5eba78da5b0f1ae6b7add388	Actinobacteria	Actinomyces	uncultured bacterium	-1,448280461	0,009230337
780f28af26e296622b43382fb709f22d	Actinobacteria	Rothia	uncultured bacterium	-2,600082463	0,002906751
80f8e6274aafab9eac94bc26710d5f35	Proteobacteria	Burkholderia-Caballeronia-Paraburkholderia	Burkholderia multivorans	13,05697744	1,46954E-22
81e4bba5db10a621b8b111733f14ef45	Proteobacteria	uncultured bacterium	uncultured bacterium	-1,54524059	0,005894317
858c875044ffb6366ddb83a10cfeef12	Firmicutes	Peptoniphilus	uncultured bacterium	3,627970651	0,000149217
8bed26e8e0ebd0021955d707cfac55ee	Firmicutes	Lactobacillus	Lactobacillus fermentum	-5,791988473	2,95E-11
92ac25e2ae0e80fceb587e96c3afad28	Proteobacteria	Rheinheimera	Unassigned	-4,114777057	3,72855E-08
954b184013e872aaf984191f0bc33b8b	Actinobacteria	Actinomyces	Unassigned	-2,310928573	0,000154329
95e44c09ed9d75a3cc550fef1a93041b	Actinobacteria	Atopobium	uncultured bacterium	-3,354538513	4,88467E-07
9ac18d4675f49b644622fcb407ae66fc	Unassigned	Unassigned	Unassigned	-1,47629874	0,00759964
9dd256bab2fce412ba79edd422bcfcd1	Firmicutes	Lactobacillus	Lactobacillus rhamnosus	-6,307041626	2,51974E-10
9fc883c2e6148f0d914af88484356917	Proteobacteria	Stenotrophomonas	Unassigned	2,628403847	0,003235178
a274261f845de19fc4a463a1da99cf16	Firmicutes	Lactobacillus	Unassigned	-3,502830776	1,22522E-05
a5ec424299f6bc17f5bfd9c468ebea6e	Actinobacteria	Actinomyces	uncultured bacterium	2,429054469	0,002906751
a7db407113fc58457468639c42048357	Actinobacteria	Corynebacterium 1	uncultured bacterium	6,991952275	3,47245E-10

b238d30677f72452169712d5bab220e3	Bacteroidetes	OLB8	Unassigned	-1,81252867	0,000645879
b4beeacc363cb59168b18188e75a09fe	Bacteroidetes	Prevotella 7	uncultured bacterium	-6,850829865	3,72855E-08
b4d4dbb9f1efcf2d9e7969d9a94163e8	Actinobacteria	Actinomyces	uncultured bacterium	2,399718513	0,007357525
baea87f07637c1c74ffaadbe314a8ec0	Fusobacteria	Fusobacterium	Unassigned	4,177841478	9,43396E-06
bcfb05a323899446406840f5700d6406	Actinobacteria	Actinomyces	uncultured bacterium	4,009616014	1,64887E-05
c190639e3a4b3fb4601b17c208a3c584	Bacteroidetes	Prevotella	uncultured bacterium	-2,672721601	4,54341E-05
c4f915300062cc9466c3111ce0d222ed	Unassigned	Unassigned	Unassigned	-2,882777428	1,14128E-06
cfb1563deb8ef0ab2d09202ba7eafed5	Firmicutes	Streptococcus	Streptococcus equinus	-3,674274821	1,60576E-06
db5ca2ae9c9907b322474e8cb5239adb	Firmicutes	Veillonella	uncultured bacterium	2,645894154	0,009142702
dcb1d9140c96abf3e102defb05564b42	Firmicutes	Veillonella	uncultured bacterium	2,687125098	0,00239054
e0831855758d1792fcd0b18eef717796	Firmicutes	Enterococcus	Unassigned	5,289017195	4,54341E-05
e50cff1ba4858bf1cfb4bf01b4566953	Firmicutes	Megasphaera	Unassigned	-3,698948401	0,000842752
f9d154d3ed332a006cf17953390db7ea	Tenericutes	Mycoplasma	Unassigned	3,963260435	2,23566E-05
fba008bc1eac2a94e22556b077fd1379	Proteobacteria	Vibrio	Unassigned	-3,925727114	1,94509E-06
ff1c417dc3af18107ffb2b87cdd1f5e6	Actinobacteria	Actinomyces	uncultured bacterium	5,122164854	1,68756E-06
