# Supplementary material for: Divergent airway microbiomes in lung transplant recipients with or without pulmonary infection
Source: Respir Res. 2021 Apr 23;22:118. doi: 10.1186/s12931-021-01724-w (PMC8063417; doi:10.1186/s12931-021-01724-w)
Supplement: Supplementary file 10 — Additional file 10: Table S4. Enrichment analysis for the three inflammatory biomarkers. [file 12931_2021_1724_MOESM10_ESM.pdf]

Additional table 4: The three attached tables contain the enrichment analysis for the three inflammatory biomarkers

Sub table 1: HBP

If the log2FoldChange is above ZERO, meaning that the ASVs were more abundant in HBP=High patients and below ZERO is more abundant in HBP=Low

ASV_ID	Phylum	Genus	Species	log2FoldChange	padj
0452d0b6f9260793becd0e37f209cc7c	Patescibacteria	uncultured bacterium	uncultured bacterium	-3,968731111	7,6716E-08
07fc17a49229a39ea32853a758254455	Firmicutes	Enterococcus	Enterococcus faecium	1,991832846	0,002823997
0834d300bbf5fa7e9eda32ee071b8c72	Actinobacteria	Cutibacterium	uncultured bacterium	-2,192204464	0,000297968
0a8a1779091d86f5a8ed2c222fc79c19	Actinobacteria	Rothia	uncultured bacterium	-5,629464566	3,17308E-12
0b18ca83ef493094b8d6965fdd13d3ad	Actinobacteria	Atopobium	uncultured bacterium	-4,680631945	3,70007E-09
0b2ba1171edfcebfe8f1f90f1a0cf866	Actinobacteria	Actinomyces	uncultured bacterium	-2,348058018	0,000168469
0bc2ff6e68ca5e252d55825929ba4201	Epsilonbacteraeota	Campylobacter	Unassigned	-2,221587997	0,000262698
0deffeaecf1c31a451ce62006c8f688e	Bacteroidetes	Prevotella 7	uncultured bacterium	2,46966207	0,000645267
11b41270aa0f2ee0cb25acad36d397e9	Proteobacteria	Shewanella	Unassigned	-5,26853141	1,8249E-14
135de8c9146a83d2572df6408f5283d9	Cyanobacteria	Unassigned	Unassigned	-2,451281096	5,58392E-05
1910dcf6315135f7947c373ec31f82f6	Firmicutes	Streptococcus	uncultured bacterium	3,394338732	6,89767E-06
22f1c5d4e53e8c961dd73e244503e7b7	Firmicutes	Streptococcus	uncultured bacterium	-3,422214096	0,00087071
25a0549b6580dfe6742eba8263d5fc63	Firmicutes	Lachnoanaerobaculum	uncultured bacterium	-2,440668218	0,000875671
2679ce9192f4b9a7fac20960748935b8	Proteobacteria	Rhodoferax	uncultured bacterium	-1,481336772	0,006107321
297d5e708fddc3355fd49ab3a8057c05	Proteobacteria	Neisseria	uncultured bacterium	-5,43124315	3,14806E-11
2b3e246dc92bce326698cc60a8608a2f	Bacteroidetes	Alloprevotella	uncultured bacterium	-6,191769143	1,59455E-11
2e5242ae8c79e05740b498c1233602ac	Unassigned	Unassigned	Unassigned	-3,633033641	3,33567E-07
3041019246205852cbbcce91bef2fef8	Firmicutes	Megasphaera	Unassigned	2,718431414	0,000181889
31ad616c6e66a96867c8fe54123f271d	Firmicutes	Veillonella	Unassigned	4,243680568	9,24954E-08
332596763b446086a5eba3746698aa6b	Firmicutes	Gemella	uncultured bacterium	-5,345467857	2,94837E-12
33f1205eacafe6210d21e955135800d6	Firmicutes	Streptococcus	Streptococcus equinus	-4,886433534	2,79431E-13
34cc9d0ff9bc451e9da9156b90906df6	Bacteroidetes	Porphyromonas	uncultured bacterium	-2,135365255	0,000619644
37145125b38f2d359d804407a13c57aa	Actinobacteria	Actinomyces	uncultured bacterium	-3,067503269	5,39219E-06
375c325427cc0f56ea2c6679b9c98ab7	Unassigned	Unassigned	Unassigned	-1,53044791	0,005408128
38b502eac93359dc19677061e0b4e889	Actinobacteria	Rothia	uncultured bacterium	-2,460209867	0,000102952
3a4871ff42be17e701fdc37a7bff65d3	Fusobacteria	Leptotrichia	uncultured bacterium	-1,916285051	0,001477667
51ff2a2903c953b84962d2fcf0905561	Unassigned	Unassigned	Unassigned	1,589378912	0,002912471
539928f136b1ee918ab61eee7e01369a	Bacteroidetes	Capnocytophaga	uncultured bacterium	-3,305409023	3,37067E-06
5615e30e748e49f0e601047d67c6a73e	Proteobacteria	Neisseria	uncultured bacterium	-2,857355699	0,001884748
56e670aa8dd0635387b32408f9c7ce3f	Bacteroidetes	Capnocytophaga	uncultured bacterium	-2,341771347	0,000262698
5818cd73593b458018d6941c486dcbe3	Firmicutes	Solobacterium	Unassigned	-3,11583965	6,89767E-06
58be7fff0121d20b6c908108fb0f5e37	Epsilonbacteraeota	Campylobacter	Unassigned	-3,15717977	0,000541176
5a80a4e82d801813fc907c456c1cf6db	Firmicutes	Ruminococcaceae UCG-014	uncultured bacterium	1,883973848	0,00566981
5de00951258e3e0a849636ad65f0628f	Bacteroidetes	Prevotella 7	uncultured bacterium	-3,94914771	9,62489E-08

64f64f158cf671861b380e95591c1d98	Firmicutes	Staphylococcus	Staphylococcus aureus	5,755821284	1,35951E-08
657fbefb76be610c3ff82c16585022a3	Firmicutes	Veillonella	uncultured bacterium	-4,911223173	1,40972E-11
67399154870db98b52be2dcd8724ef86	Actinobacteria	Salinibacterium	Unassigned	-1,690767332	0,006785224
6c247835d06811be636d8bffcf005b3d	Actinobacteria	Actinomyces	Unassigned	-2,085375244	0,004334391
6ddebcc029eb24f1ca349b7345747970	Bacteroidetes	Prevotella 7	uncultured bacterium	3,054768324	3,46638E-05
6fb82033ffa11adc2a7cc76c3518d960	Proteobacteria	Psychrobacter	Unassigned	-3,974070041	1,00426E-08
72d8f60e7a30d05555a261cbd3f8a6d4	Firmicutes	Selenomonas 3	uncultured bacterium	-4,453274303	3,14371E-10
75a653fc99420b77c4dedb9c5451b64f	Firmicutes	Streptococcus	uncultured bacterium	-2,47315806	0,00015231
780f28af26e296622b43382fb709f22d	Actinobacteria	Rothia	uncultured bacterium	-2,881951392	0,000172253
80bf3a5426b64c700d7f90abf6fcd2c7	Firmicutes	Streptococcus	Unassigned	-3,2011654	1,40382E-05
80f8e6274aafab9eac94bc26710d5f35	Proteobacteria	Burkholderia-Caballeronia-Paraburkholderia	Burkholderia multivorans	13,31049743	6,71041E-31
858c875044ffb6366ddb83a10cfeef12	Firmicutes	Peptoniphilus	uncultured bacterium	3,862588995	2,86762E-06
8e0c3981719553595d5256a6605ffa8a	Firmicutes	Streptococcus	uncultured bacterium	-2,840000673	0,000976958
92ac25e2ae0e80fceb587e96c3afad28	Proteobacteria	Rheinheimera	Unassigned	-4,203378795	3,04053E-09
92e84f9323f8817fbf5f2fbff70db883	Fusobacteria	Leptotrichia	Unassigned	-1,888628028	0,0009799
950529b0f084e686648a10c19a32f05f	Proteobacteria	Pseudomonas	uncultured bacterium	-1,923274496	0,0009799
954b184013e872aaf984191f0bc33b8b	Actinobacteria	Actinomyces	Unassigned	-1,989076072	0,001073213
95e44c09ed9d75a3cc550fef1a93041b	Actinobacteria	Atopobium	uncultured bacterium	-3,54632065	1,5456E-07
95f1a13532c3412d29e2d86ed7a5d6de	Firmicutes	Oribacterium	uncultured bacterium	-2,701167131	1,63006E-05
9867e986a56d503728dd0be56e9a0bd8	Firmicutes	Stomatobaculum	uncultured bacterium	-3,071530621	1,48515E-06
9fc883c2e6148f0d914af88484356917	Proteobacteria	Stenotrophomonas	Unassigned	2,843871303	0,000147185
a29d7e4aa55d8db376ee2fc4a026e248	Cyanobacteria	Unassigned	Unassigned	-3,014318779	1,07346E-05
a5ec424299f6bc17f5bfd9c468ebea6e	Actinobacteria	Actinomyces	uncultured bacterium	-2,67782984	2,20232E-05
a7db407113fc58457468639c42048357	Actinobacteria	Corynebacterium 1	uncultured bacterium	7,243696896	7,04252E-14
b238d30677f72452169712d5bab220e3	Bacteroidetes	OLB8	Unassigned	-1,527446845	0,005176667
b4d4dbb9f1efcf2d9e7969d9a94163e8	Actinobacteria	Actinomyces	uncultured bacterium	2,608478196	0,000389393
b55641bd1ab1364165b51136cc2ac924	Proteobacteria	Haemophilus	uncultured bacterium	-3,111921476	0,000227249
b8573cd0f3f8b8d0abbcb1acce93f516	Firmicutes	Lachnoanaerobaculum	Unassigned	-1,529517437	0,005176667
bcfb05a323899446406840f5700d6406	Actinobacteria	Actinomyces	uncultured bacterium	-3,220324825	0,000305665
c190639e3a4b3fb4601b17c208a3c584	Bacteroidetes	Prevotella	uncultured bacterium	-5,396810198	3,25831E-12
c48183b0aa6aebe369bd269eec454d1a	Bacteroidetes	Prevotella	uncultured bacterium	-6,282170736	3,86587E-13
c86abaab9cb3bd87ae21d837179b8f35	Proteobacteria	Halomonas	Unassigned	-1,77918617	0,000930472
cd7ef64166d4ca3a97f7ac768923b3f7	Firmicutes	Streptococcus	uncultured bacterium	-4,535129613	8,25459E-10
cfb1563deb8ef0ab2d09202ba7eafed5	Firmicutes	Streptococcus	Streptococcus equinus	-4,565174426	1,40972E-11
d0e03e47cd685455ea943184509b5283	Firmicutes	Gemella	uncultured bacterium	-3,578388122	0,000305665
d119718ecc20c6da709c5a2f6cd961ee	Epsilonbacteraeota	Campylobacter	Unassigned	-2,270105561	0,000300762
d2c0e30f246642775c7056a330db7629	Firmicutes	Oribacterium	uncultured bacterium	-3,216987985	2,90883E-06
dbcc706b3b209681069dbf596f41ef78	Firmicutes	Granulicatella	Unassigned	-2,752229288	6,89767E-06
dcb1d9140c96abf3e102defb05564b42	Firmicutes	Veillonella	uncultured bacterium	2,904091987	9,21167E-05
dcd4fdd8560743aefb1d01fa31a24364	Actinobacteria	Actinomyces	Unassigned	2,802835083	0,000195907

defe218786823b755e3195ad9c10224b	Actinobacteria	Actinomyces	Unassigned	-1,552250709	0,006655497
e0831855758d1792fcd0b18eef717796	Firmicutes	Enterococcus	Unassigned	10,69021291	1,59449E-21
e50cff1ba4858bf1cfb4bf01b4566953	Firmicutes	Megasphaera	Unassigned	-2,651776206	0,000630304
efe692b2c861de453fe581761ae03129	Fusobacteria	Leptotrichia	uncultured bacterium	-2,143464737	0,000604173
f1d7011789d24af98b72583e81b754cd	Proteobacteria	Unassigned	Unassigned	-3,424477816	1,09111E-06
f9b0a8ac4ae3575e90ea1cc796a61a34	Firmicutes	Staphylococcus	Unassigned	4,516077317	3,46399E-05
f9d154d3ed332a006cf17953390db7ea	Tenericutes	Mycoplasma	Unassigned	3,608658877	3,73857E-06
fba008bc1eac2a94e22556b077fd1379	Proteobacteria	Vibrio	Unassigned	-4,728820576	4,1416E-11
ff1c417dc3af18107ffb2b87cdd1f5e6	Actinobacteria	Actinomyces	uncultured bacterium	-4,378689257	1,80278E-09

Sub table 2: IL_1B

If the log2FoldChange is above ZERO, meaning that the ASVs were more abundant in IL_1b=High patients and below ZERO is more abundant in IL_1b=Low

**ASV_ID**	**Phylum**	**Genus**	**Species**	**log2FoldChange**	**padj**
0452d0b6f9260793becd0e37f209cc7c	Patescibacteria	uncultured bacterium	uncultured bacterium	-4,163602624	1,22648E-08
0834d300bbf5fa7e9eda32ee071b8c72	Actinobacteria	Cutibacterium	uncultured bacterium	-2,365334618	0,000129631
0bc2ff6e68ca5e252d55825929ba4201	Epsilonbacteraeota	Campylobacter	Unassigned	-2,479977573	4,26687E-05
0deffeaecf1c31a451ce62006c8f688e	Bacteroidetes	Prevotella 7	uncultured bacterium	2,304027456	0,005446451
11b41270aa0f2ee0cb25acad36d397e9	Proteobacteria	Shewanella	Unassigned	-5,506014895	6,35621E-17
135de8c9146a83d2572df6408f5283d9	Cyanobacteria	Unassigned	Unassigned	-2,719228859	5,42989E-06
1910dcf6315135f7947c373ec31f82f6	Firmicutes	Streptococcus	uncultured bacterium	2,203893728	0,005737708
22f1c5d4e53e8c961dd73e244503e7b7	Firmicutes	Streptococcus	uncultured bacterium	-4,334031684	5,70107E-05
25a0549b6580dfe6742eba8263d5fc63	Firmicutes	Lachnoanaerobaculum	uncultured bacterium	-2,228056391	0,00592473
2679ce9192f4b9a7fac20960748935b8	Proteobacteria	Rhodoferax	uncultured bacterium	-1,695664481	0,001493424
297d5e708fddc3355fd49ab3a8057c05	Proteobacteria	Neisseria	uncultured bacterium	-5,746404586	6,78427E-13
3041019246205852cbbcce91bef2fef8	Firmicutes	Megasphaera	Unassigned	2,54657815	0,001977094
33f1205eacafe6210d21e955135800d6	Firmicutes	Streptococcus	Streptococcus equinus	-5,177066884	6,4929E-12
37145125b38f2d359d804407a13c57aa	Actinobacteria	Actinomyces	uncultured bacterium	-3,354422965	4,69716E-07
375c325427cc0f56ea2c6679b9c98ab7	Unassigned	Unassigned	Unassigned	-2,091416234	5,70107E-05
3a4871ff42be17e701fdc37a7bff65d3	Fusobacteria	Leptotrichia	uncultured bacterium	-2,159326889	0,000341821
3fb9b3d2d38160ef0a83dfb7fe9c3f9c	Firmicutes	Veillonella	uncultured bacterium	-1,386296901	0,005937775
4fa7999ddeac300066f4e86af2e94fc6	Bacteroidetes	Bergeyella	uncultured bacterium	-2,839289954	0,002102627
4fd9aec3a60775f87fb4560b99c27290	Actinobacteria	Actinomyces	uncultured bacterium	-2,243704588	0,001695291
539928f136b1ee918ab61eee7e01369a	Bacteroidetes	Capnocytophaga	uncultured bacterium	-3,639774251	2,19164E-07
5615e30e748e49f0e601047d67c6a73e	Proteobacteria	Neisseria	uncultured bacterium	-2,719711769	0,007576669
56e670aa8dd0635387b32408f9c7ce3f	Bacteroidetes	Capnocytophaga	uncultured bacterium	-2,605386057	4,88686E-05
5818cd73593b458018d6941c486dcbe3	Firmicutes	Solobacterium	Unassigned	-3,403915501	7,47919E-07
58be7fff0121d20b6c908108fb0f5e37	Epsilonbacteraeota	Campylobacter	Unassigned	-3,081644517	0,001912792
5de00951258e3e0a849636ad65f0628f	Bacteroidetes	Prevotella 7	uncultured bacterium	-4,252162784	5,02081E-09

64f64f158cf671861b380e95591c1d98	Firmicutes	Staphylococcus	Staphylococcus aureus	5,152072904	4,55726E-06
657fbefb76be610c3ff82c16585022a3	Firmicutes	Veillonella	uncultured bacterium	-5,144151974	5,68613E-13
666c842b3a3a5756feed9bb2856d76fc	Proteobacteria	Psychrobacter	Unassigned	-1,854461153	0,005289134
67024a6c5e922e807b1d4841e24777fe	Bacteroidetes	Prevotella 7	uncultured bacterium	-7,304164829	1,03161E-12
67399154870db98b52be2dcd8724ef86	Actinobacteria	Salinibacterium	Unassigned	-1,97432342	0,001744038
6c247835d06811be636d8bffcf005b3d	Actinobacteria	Actinomyces	Unassigned	-4,478030275	7,7645E-08
6fb82033ffa11adc2a7cc76c3518d960	Proteobacteria	Psychrobacter	Unassigned	-3,524688225	1,67113E-06
72d8f60e7a30d05555a261cbd3f8a6d4	Firmicutes	Selenomonas 3	uncultured bacterium	-4,761890435	2,70877E-12
75a653fc99420b77c4dedb9c5451b64f	Firmicutes	Streptococcus	uncultured bacterium	-2,741898621	2,68046E-05
780f28af26e296622b43382fb709f22d	Actinobacteria	Rothia	uncultured bacterium	-2,96620337	0,000129631
80bf3a5426b64c700d7f90abf6fcd2c7	Firmicutes	Streptococcus	Unassigned	-4,654946634	5,9593E-10
80f8e6274aafab9eac94bc26710d5f35	Proteobacteria	Burkholderia-Caballeronia-Paraburkholderia	Burkholderia multivorans	13,10523327	4,21521E-24
81e4bba5db10a621b8b111733f14ef45	Proteobacteria	uncultured bacterium	uncultured bacterium	-1,483721561	0,00420216
858c875044ffb6366ddb83a10cfeef12	Firmicutes	Peptoniphilus	uncultured bacterium	3,672517258	6,52004E-05
875b5539560501dfcf2a0498403a887e	Firmicutes	[Eubacterium] nodatum group	Unassigned	-1,575510098	0,00420216
8e0c3981719553595d5256a6605ffa8a	Firmicutes	Streptococcus	uncultured bacterium	-3,899505412	3,40925E-05
92ac25e2ae0e80fceb587e96c3afad28	Proteobacteria	Rheinheimera	Unassigned	-4,599786605	1,39774E-11
954b184013e872aaf984191f0bc33b8b	Actinobacteria	Actinomyces	Unassigned	-2,236079349	0,000239053
95e44c09ed9d75a3cc550fef1a93041b	Actinobacteria	Atopobium	uncultured bacterium	-3,843220848	5,02081E-09
95f1a13532c3412d29e2d86ed7a5d6de	Firmicutes	Oribacterium	uncultured bacterium	-2,348360604	0,000345697
a274261f845de19fc4a463a1da99cf16	Firmicutes	Lactobacillus	Unassigned	3,343140854	0,000672935
a29d7e4aa55d8db376ee2fc4a026e248	Cyanobacteria	Unassigned	Unassigned	-3,293926966	1,45104E-06
a7db407113fc58457468639c42048357	Actinobacteria	Corynebacterium 1	uncultured bacterium	7,03985594	2,74791E-11
b238d30677f72452169712d5bab220e3	Bacteroidetes	OLB8	Unassigned	-1,745232001	0,001211809
b4d4dbb9f1efcf2d9e7969d9a94163e8	Actinobacteria	Actinomyces	uncultured bacterium	2,439271518	0,003648179
b55641bd1ab1364165b51136cc2ac924	Proteobacteria	Haemophilus	uncultured bacterium	4,310510211	6,52004E-05
b8573cd0f3f8b8d0abbcb1acce93f516	Firmicutes	Lachnoanaerobaculum	Unassigned	-1,747482886	0,001211809
c190639e3a4b3fb4601b17c208a3c584	Bacteroidetes	Prevotella	uncultured bacterium	-5,174395321	1,16954E-11
c48183b0aa6aebe369bd269eec454d1a	Bacteroidetes	Prevotella	uncultured bacterium	-6,600332117	4,72755E-15
c68c23304236005874b0871dbb81ad23	Actinobacteria	Actinomyces	uncultured bacterium	-3,588170345	0,000351353
c86abaab9cb3bd87ae21d837179b8f35	Proteobacteria	Halomonas	Unassigned	-2,014133219	0,000129631
cd7ef64166d4ca3a97f7ac768923b3f7	Firmicutes	Streptococcus	uncultured bacterium	-6,430683931	9,20997E-17
cfb1563deb8ef0ab2d09202ba7eafed5	Firmicutes	Streptococcus	Streptococcus equinus	-4,928381479	1,48965E-14
d119718ecc20c6da709c5a2f6cd961ee	Epsilonbacteraeota	Campylobacter	Unassigned	-1,950866599	0,003607069
d2c0e30f246642775c7056a330db7629	Firmicutes	Oribacterium	uncultured bacterium	-2,418824409	0,002142671
db5ca2ae9c9907b322474e8cb5239adb	Firmicutes	Veillonella	uncultured bacterium	3,685150782	3,40925E-05
dcb1d9140c96abf3e102defb05564b42	Firmicutes	Veillonella	uncultured bacterium	3,12082497	0,000204519
dcd4fdd8560743aefb1d01fa31a24364	Actinobacteria	Actinomyces	Unassigned	3,605383157	4,26687E-05
e4dc155aa6acf224412546f84bab7b20	Actinobacteria	Actinomyces	Unassigned	2,479822437	0,003277095
e50cff1ba4858bf1cfb4bf01b4566953	Firmicutes	Megasphaera	Unassigned	-3,070338575	9,07752E-05

f1d7011789d24af98b72583e81b754cd	Proteobacteria	Unassigned	Unassigned	-2,969676663	9,03575E-05
f68dfd1268b4693aeadd5c5cd01e4c3f	Firmicutes	Staphylococcus	uncultured bacterium	-1,327440576	0,009075953
f6cec6cce9b8cbab9e59d0fe1316fd2b	Actinobacteria	Rothia	uncultured bacterium	-3,146506381	0,001977094
f9b0a8ac4ae3575e90ea1cc796a61a34	Firmicutes	Staphylococcus	Unassigned	4,718102824	0,000129631
f9d154d3ed332a006cf17953390db7ea	Tenericutes	Mycoplasma	Unassigned	3,421482906	9,07752E-05
fba008bc1eac2a94e22556b077fd1379	Proteobacteria	Vibrio	Unassigned	-5,167952833	5,32526E-14
ff1c417dc3af18107ffb2b87cdd1f5e6	Actinobacteria	Actinomyces	uncultured bacterium	-2,836883589	1,92688E-06

Sub table 3: IL_8

If the log2FoldChange is above ZERO, meaning that the ASVs were more abundant in IL_8=High patients and below ZERO is more abundant in IL_8=Low

ASV_ID	Phylum	Genus	Species	log2FoldChange	padj
0a8a1779091d86f5a8ed2c222fc79c19	Actinobacteria	Rothia	uncultured bacterium	-3,833824265	5,45916E-05
0deffeaecf1c31a451ce62006c8f688e	Bacteroidetes	Prevotella 7	uncultured bacterium	2,265556764	0,009357357
11b41270aa0f2ee0cb25acad36d397e9	Proteobacteria	Shewanella	Unassigned	-3,95692156	5,93779E-07
135de8c9146a83d2572df6408f5283d9	Cyanobacteria	Unassigned	Unassigned	-1,917575069	0,004516627
1408b62055d449b2abb904abee855875	Firmicutes	Veillonella	Unassigned	4,795191984	5,93779E-07
1910dcf6315135f7947c373ec31f82f6	Firmicutes	Streptococcus	uncultured bacterium	3,167119579	0,000355409
22f1c5d4e53e8c961dd73e244503e7b7	Firmicutes	Streptococcus	uncultured bacterium	-3,313121209	0,006947994
25a0549b6580dfe6742eba8263d5fc63	Firmicutes	Lachnoanaerobaculum	uncultured bacterium	-2,348949677	0,00424755
2679ce9192f4b9a7fac20960748935b8	Proteobacteria	Rhodoferax	uncultured bacterium	-1,761736393	0,000900282
297d5e708fddc3355fd49ab3a8057c05	Proteobacteria	Neisseria	uncultured bacterium	-5,838394723	2,99402E-13
3041019246205852cbbcce91bef2fef8	Firmicutes	Megasphaera	Unassigned	2,50662536	0,003704347
33f1205eacafe6210d21e955135800d6	Firmicutes	Streptococcus	Streptococcus equinus	-4,663354337	1,57121E-08
37145125b38f2d359d804407a13c57aa	Actinobacteria	Actinomyces	uncultured bacterium	-3,439361824	3,01964E-07
375c325427cc0f56ea2c6679b9c98ab7	Unassigned	Unassigned	Unassigned	-1,739284982	0,002674522
38b502eac93359dc19677061e0b4e889	Actinobacteria	Rothia	uncultured bacterium	-4,000893401	2,79732E-06
3fb9b3d2d38160ef0a83dfb7fe9c3f9c	Firmicutes	Veillonella	uncultured bacterium	-1,445742875	0,003760216
4fd9aec3a60775f87fb4560b99c27290	Actinobacteria	Actinomyces	uncultured bacterium	-2,34675944	0,00114565
539928f136b1ee918ab61eee7e01369a	Bacteroidetes	Capnocytophaga	uncultured bacterium	-3,735175768	1,40126E-07
56e670aa8dd0635387b32408f9c7ce3f	Bacteroidetes	Capnocytophaga	uncultured bacterium	-2,684374825	3,19255E-05
5818cd73593b458018d6941c486dcbe3	Firmicutes	Solobacterium	Unassigned	-3,489142109	4,73045E-07
64f64f158cf671861b380e95591c1d98	Firmicutes	Staphylococcus	Staphylococcus aureus	5,02607895	1,71774E-05
657fbefb76be610c3ff82c16585022a3	Firmicutes	Veillonella	uncultured bacterium	-2,886698593	0,000130645
67024a6c5e922e807b1d4841e24777fe	Bacteroidetes	Prevotella 7	uncultured bacterium	-5,128936629	1,9809E-05
67399154870db98b52be2dcd8724ef86	Actinobacteria	Salinibacterium	Unassigned	-2,056727191	0,001244544
6c247835d06811be636d8bffcf005b3d	Actinobacteria	Actinomyces	Unassigned	-4,603951725	4,85738E-08
6fb82033ffa11adc2a7cc76c3518d960	Proteobacteria	Psychrobacter	Unassigned	-3,364516302	8,621E-06
75a653fc99420b77c4dedb9c5451b64f	Firmicutes	Streptococcus	uncultured bacterium	-2,822202067	1,64866E-05

780f28af26e296622b43382fb709f22d	Actinobacteria	Rothia	uncultured bacterium	-3,083709211	9,716E-05
80f8e6274aafab9eac94bc26710d5f35	Proteobacteria	Burkholderia-Caballeronia-Paraburkholderia	Burkholderia multivorans	12,69831386	1,31084E-20
81e4bba5db10a621b8b111733f14ef45	Proteobacteria	uncultured bacterium	uncultured bacterium	-1,545448364	0,002674522
858c875044ffb6366ddb83a10cfeef12	Firmicutes	Peptoniphilus	uncultured bacterium	3,628182287	0,000162908
875b5539560501dfcf2a0498403a887e	Firmicutes	[Eubacterium] nodatum group	Unassigned	-1,639168157	0,002831644
896556c6f732829f8fd14a98aac59cda	Patescibacteria	uncultured bacterium	uncultured bacterium	-1,336195039	0,007020408
8e0c3981719553595d5256a6605ffa8a	Firmicutes	Streptococcus	uncultured bacterium	-3,967366156	3,19255E-05
92ac25e2ae0e80fceb587e96c3afad28	Proteobacteria	Rheinheimera	Unassigned	-4,709204136	5,0629E-12
954b184013e872aaf984191f0bc33b8b	Actinobacteria	Actinomyces	Unassigned	-2,310753945	0,000182978
95e44c09ed9d75a3cc550fef1a93041b	Actinobacteria	Atopobium	uncultured bacterium	-3,354409772	3,60262E-07
a274261f845de19fc4a463a1da99cf16	Firmicutes	Lactobacillus	Unassigned	3,227210275	0,001787087
a29d7e4aa55d8db376ee2fc4a026e248	Cyanobacteria	Unassigned	Unassigned	-2,68107711	0,000355409
a373695db3432a96e70d8b0c4a4b520a	Fusobacteria	Fusobacterium	uncultured bacterium	4,584007879	1,61559E-06
a37c24531a8db843e4eb475bc8624e4e	Actinobacteria	Candidatus Aquiluna	uncultured bacterium	-1,373621445	0,004498242
a5ec424299f6bc17f5bfd9c468ebea6e	Actinobacteria	Actinomyces	uncultured bacterium	2,38321116	0,002990215
a7db407113fc58457468639c42048357	Actinobacteria	Corynebacterium 1	uncultured bacterium	6,992153514	2,24356E-10
b238d30677f72452169712d5bab220e3	Bacteroidetes	OLB8	Unassigned	-1,812249063	0,000748288
b4d4dbb9f1efcf2d9e7969d9a94163e8	Actinobacteria	Actinomyces	uncultured bacterium	2,399951737	0,006356709
b55641bd1ab1364165b51136cc2ac924	Proteobacteria	Haemophilus	uncultured bacterium	4,181773297	0,000201374
c190639e3a4b3fb4601b17c208a3c584	Bacteroidetes	Prevotella	uncultured bacterium	-5,265580433	5,9983E-12
c48183b0aa6aebe369bd269eec454d1a	Bacteroidetes	Prevotella	uncultured bacterium	-6,693063607	1,40822E-15
c68c23304236005874b0871dbb81ad23	Actinobacteria	Actinomyces	uncultured bacterium	-3,725812564	0,00027209
c86abaab9cb3bd87ae21d837179b8f35	Proteobacteria	Halomonas	Unassigned	-1,753627306	0,001588804
cd7ef64166d4ca3a97f7ac768923b3f7	Firmicutes	Streptococcus	uncultured bacterium	-6,621170132	2,76054E-19
cfb1563deb8ef0ab2d09202ba7eafed5	Firmicutes	Streptococcus	Streptococcus equinus	-5,030510728	1,45065E-15
d119718ecc20c6da709c5a2f6cd961ee	Epsilonbacteraeota	Campylobacter	Unassigned	-2,038774966	0,002674522
db5ca2ae9c9907b322474e8cb5239adb	Firmicutes	Veillonella	uncultured bacterium	4,359910312	1,79028E-06
dcb1d9140c96abf3e102defb05564b42	Firmicutes	Veillonella	uncultured bacterium	3,078217085	0,000472984
dcd4fdd8560743aefb1d01fa31a24364	Actinobacteria	Actinomyces	Unassigned	3,561220824	0,000101956
e0831855758d1792fcd0b18eef717796	Firmicutes	Enterococcus	Unassigned	10,40699797	5,48471E-16
e4dc155aa6acf224412546f84bab7b20	Actinobacteria	Actinomyces	Unassigned	2,440253554	0,005668688
f1d7011789d24af98b72583e81b754cd	Proteobacteria	Unassigned	Unassigned	-2,503548455	0,002674522
f6cec6cce9b8cbab9e59d0fe1316fd2b	Actinobacteria	Rothia	uncultured bacterium	-4,872707664	1,09931E-06
f9b0a8ac4ae3575e90ea1cc796a61a34	Firmicutes	Staphylococcus	Unassigned	4,766744011	0,000189238
f9d154d3ed332a006cf17953390db7ea	Tenericutes	Mycoplasma	Unassigned	3,377848738	0,000216674
fba008bc1eac2a94e22556b077fd1379	Proteobacteria	Vibrio	Unassigned	-4,877529643	1,11878E-11
ff1c417dc3af18107ffb2b87cdd1f5e6	Actinobacteria	Actinomyces	uncultured bacterium	3,805981322	0,000820332
